# Prognostic factors in metastatic nasopharyngeal carcinoma

**DOI:** 10.1016/j.bjorl.2020.05.022

**Published:** 2020-07-04

**Authors:** Nabil Toumi, Sana Ennouri, Ilhem Charfeddine, Jamel Daoud, Afef Khanfir

**Affiliations:** a University of Sfax, Habib Bourguiba Hospital, Department of Medical Oncology, Sfax, Tunisia; b University of Sfax, Habib Bourguiba Hospital, Department of Otorhinolaryngology Head and Neck Surgery, Sfax, Tunisia; c University of Sfax, Habib Bourguiba Hospital, Department of Radiotherapy, Sfax, Tunisia

**Keywords:** Nasopharyngeal cancer, Metastasis, Prognosis

## Abstract

**Introduction:**

Nasopharyngeal carcinoma has the highest metastatic potential of all head and neck cancers. The survival time of patients with nasopharyngeal carcinoma has improved significantly in the last decades due to the use of combination of chemotherapy and radiotherapy, as well as advances in radiotherapy techniques. However, appropriately 30% of patients with nasopharyngeal carcinoma suffer a poor prognosis, mainly due to distant metastasis.

**Objective:**

The study aimed to identify the survival and prognostic factors in metastatic nasopharyngeal carcinoma.

**Methods:**

A retrospective analysis was conducted in patients treated for synchronous metastatic nasopharyngeal carcinoma or metachronous metastatic nasopharyngeal carcinoma for 14 years (2003–2016). Overall survival was analyzed using the Kaplan-Meier method and compared using the log-rank test for the whole population and both groups of patients. Multivariate analysis was performed using the Cox model; *p*-values < 0.05 were considered to indicate statistical significance.

**Results:**

One hundred and twelve patients with metastatic nasopharyngeal carcinoma were included (51 patients with metastatic nasopharyngeal carcinoma, and 61 patients with metachronous metastatic nasopharyngeal carcinoma). In the whole population, the median overall survival was 10 months (1–156 months). In the multivariate analysis, female gender, poor performance status (WHO > 1) and metachronous metastasis were independent prognostic factors. In the metastatic nasopharyngeal carcinoma patients, the median overall survival was 13 months (1–156 months). In multivariate analysis, independent prognostic factors were non-oligometastatic disease, severe (G3‒G4) chemotherapy toxicity and the lack of nasopharyngeal and metastatic site irradiation. In the metachronous metastatic nasopharyngeal carcinoma patients, the median overall survival was 7 months (1–41 months). In multivariate analysis, the poor performance status (WHO > 1) was an independent metastatic nasopharyngeal carcinoma prognostic factor.

**Conclusion:**

Oligometastatic patients with synchronous metastatic nasopharyngeal carcinoma had better survival. The locoregional treatment of primitive nasopharyngeal carcinoma improved survival in patients with metastatic nasopharyngeal carcinoma who responded to induction chemotherapy. Local irradiation of metastatic sites improved survival of metastatic nasopharyngeal carcinoma patients. Grade 3 or 4 chemotherapy toxicity altered survival among patients with synchronous metastatic nasopharyngeal carcinoma.

## Introduction

Nasopharyngeal carcinoma (NPC) is common in Tunisia, which is among the middle-risk countries of NPC.^1^ NPC has the highest metastatic potential of all head and neck cancers.^2^ At diagnosis, the proportion of initially metastatic patients is around 5%–11%.^3^, ^4^ During the follow-up of treated NPC, the rate of metastatic relapse is around 8%–58%.^5^, ^6^, ^7^

The survival time of patients with NPC has improved significantly in the last decades due to the use of combination of chemotherapy and radiotherapy and to advances in radiotherapy techniques. However, approximately 30% of patients with NPC suffer a poor prognosis, mainly due to distant metastasis.^8^ The overall survival (OS) of patients with synchronous metastases rarely exceeds 30 months.^3^, ^9^ In patients with metachronous metastases, OS from the date of diagnosis of metastatic disease is around 20 months.^10^, ^11^

The study aimed to identify the survival and prognostic factors of metastatic NPC in all patients with metastatic NPC and, in each group of patients with the metachronous and synchronous metastases.

## Methods

We conducted a retrospective study of patients with metastatic NPC. Between January 2003 and December 2016, 112 patients diagnosed and treated for metastatic NPC were collected and analyzed. The tumor was classified according to the seventh edition of the TNM classification by the Union for International Cancer Control. A computed tomography scan of the chest and upper abdomen and a bone scan were performed for initial staging. After treatment completion, follow-up assessments were conducted every 3 months for the first 2 years, every 6 months from the third through the fifth year, and annually thereafter. Follow-up included medical history and physical examination, and a computed tomography scan of the chest every 6–12 months. If metastases were suspected, further imaging may be required to confirm the diagnosis.

Patients with metachronous metastatic NPC (mmNPC) were patients who had an occurrence of distant metastasis after the definitive chemo-radiotherapy for NPC.

Patients with synchronous metastatic NPC (smNPC) were patients who had distant metastasis since the initial diagnosis or before the ending of the definitive chemoradiation for NPC.

The therapeutic decision was made in a multidisciplinary team (MDT) involving the medical oncologist, the radiation oncologist, the pathologist, and the head and neck surgeon. Nasopharyngeal radiation for smNPC patients was discussed by the MDT when the disease process had stabilized in the patient or partial/complete response after 3 courses of induction chemotherapy.

Demographics, comorbidities, tumor characteristics, and overall survival were recorded, as well as therapies including chemotherapy (CT) and radiation therapy (RT). Oligometastatic disease was defined by less than five metastases and/or metastases in only one site.^12^ OS was defined as the period from diagnosis of metastatic disease to death from any cause. Patients who had a long-term overall survival > 36 months were classified as long-term survivors.

Studied prognostic factors included the age, gender, performance status, T and N classification, treatment modalities and toxicity of CT.

All variables including baseline characteristics were presented as a number with a percentage for categorical variables, mean ± standard deviation for continuous variables following a normal distribution, and median with interquartile range (IQ) or extremes for continuous variables not following a normal distribution. Normal distribution was tested with the Kolmogorov-Smirnov test.

Baseline characteristics were compared using the chi2 and Mann-Whitney *U* test for categorical and continuous variables respectively.

Survival was estimated using the Kaplan-Meier method. Statistical comparison between patient subgroups survival was carried out using the log-Rank test (statistical significance was defined as *p* ≤  0.05). Multivariable analysis was performed using the Cox model (*p* ≤  0.05 was used as the cut-off value of statistical significance).

Statistical analysis used the Statistical Package for Social Sciences (SPSS) for Windows (SPSS Inc., Chicago, IL, USA) software version 20.

## Results

### Patient, tumor and treatment characteristics

During the 14 year study period, 348 patients had non- metastastatic NPC and 112 patients had metastatic NPC (51 patients with smNPC and 61 patients with mmNPC). The median age of patients was 48.7 ± 16.5 years. Male predominance was found in the whole population (77.7%) and in both smNPC (78.4%) and mmNPC (77%). Patients had a good performance status (WHO = 1) in 68.8% of cases (Table 1).

**Table 1:** Patients, tumor, and treatment characteristics of whole population and in synchronous and metachronous metastatic nasopharyngeal carcinoma.

		All patients (%)	smNPC (%)	mmNPC (%)	*p*-value
Age^a^	Mean	48.7	50.47	45.55	0.027^b^
	SD	16.5	13.4	16.85	
Gender	Male	87 (77.7)	40 (78.4)	47 (77)	0.861^b^
	Female	25 (22.3)	11 (21.6)	14 (23)	
Performance status	WHO = 1	77 (68.8)	39 (76.5)	38 (62.3)	0.107^b^
	WHO > 1	35 (31.2)	12 (23.5)	23 (37.7)	
T classification at initial diagnosis	T0/T1/T2	37 (33)	14 (27.5)	23 (37.7)	0.25^b^
	T3/T4	75 (67)	37 (72.5)	38 (62.3)	
N classification at initial diagnosis	N0/N1/N2	51 (45.5)	21 (41.2)	30 (49.2)	0.397^b^
	N3	61 (54.5)	30 (58.8)	31 (50.8)	
Oligometastatic patients	Yes	64 (57.2)	32 (62.7)	32 (52.5)	0.133^b^
	No	48 (42.8)	19 (37.3)	29 (47.5)	
CT for metastatic disease	Yes	87 (77.7)	42 (82.4)	45 (73.8)	0.27^b^
	No	25 (22.3)	9 (17.6)	16 (26.2)	
CT associated with RT of a metastatic site	Yes	15 (23.1)	6 (24)	9 (22.5)	0.01^b^
	Exclusive CT	50 (76.9)	19 (76)	31 (77.5)	

SD, Standard Deviation; WHO, WHO performance status classification; smNPC, Synchronous Metastatic Nasopharyngeal Carcinoma; mmNPC, Metachronous Metastatic Nasopharyngeal Carcinoma; CT, Chemotherapy; RT, Radiation Therapy; *p*-value: *p*-value of < 0.05 was considered statistically significant.

a Age variable follows normal distribution in whole population and in each group of patients; values are mean ± standard deviation.

b Student’s *t*-test; ^b^Chi-Squared test.

According to the initial classification, patients had a T3 or T4 tumor in 67% of cases and N3 lymph node involvement in 54.4% of cases (Table 1). The most common histological subtype was undifferentiated nasopharyngeal carcinoma (110 patients). One patient in the group of smNPC and one patient in the mmNPC had non-keratinizing carcinoma. In all patients who had initially non-metastatic disease, 3 cases had exclusive nasopharyngeal RT, 18 cases had nasopharyngeal concomitant chemoradiation, and 40 cases had induction chemotherapy. Of those latter 40 cases, further treatment was RT in 5 cases, and chemoradiation in 35 cases.

In mmNPC group, 70.5% of cases reported metastatic relapse within the first year after the end of treatment. The median time of relapse was 9 months (3‒78 months).

Bone metastasis was the most frequent metastatic site (67.9%) followed by liver (41.1%), extra-regional lymph nodes (29.5%) and lung (27.7%). Eighty-seven patients received chemotherapy for metastatic disease (42 patients with smNPC and 45 patients with mmNPC). Twenty-six patients with smNPC received nasopharyngeal RT. Six patients with smNPC and 9 patients with mmNPC received both chemotherapy, and metastatic site irradiation (Table 1).

### Prognostic factors

#### The whole population prognostic factors of survival

The median OS of the whole population was 10 months (1‒156 months). OS at 1 year, 2 years, 3 years and 5 years was respectively 40%, 14.5%, 7.2% et 1.2%. In univariate analysis, female gender (*p* =  0.018), metachronous metastasis (*p* <  0.001), the poor (WHO > 1) performance status (*p* <  0.001) and having non-oligometastatic disease (*p* = 0.006), all contributed to the decreased survival rates significantly (Table 2). In multivariate analysis, female gender (HR = 1.62 [1.01–2.58], *p* =  0.04), poor performance status (HR = 0.37 [0.24‒0.58], *p* <  0.001) and metachronous metastasis (HR = 1.86 [1.24–2.77], *p* =  0.003) were independent prognostic factors. Table 2 shows prognostic factors in the whole population in univariate and multivariate analysis.

**Table 2:** Prognostic factors of the whole population in univariate and multivariale analysis.

Prognostic factor	Univariate analysis^a^					Multivariate analysis^b^			
	n	OS at 1 year	OS at 3 years	OS at 5 years	*p*-value	HR	Low	High	*p*-value
Gender									
Male	87	42.4%	9.3%	3.1%	0.018	1.62	1.01	2.58	0.04
Female	25	32%	0%	0%					
Mean age									
≤ 48 years old	52	43.1%	7.8%	2.6%	0.56				
> 48 years old	60	37.3%	6.4%	0%					
Age									
≤ 30 years old	15	33.3%	0%	0%	0.61				
> 30 years old	97	38.9%	6.1%	1.2%					
Metastases of NPC									
Synchronous	51	56.9%	11.8%	4.7%	0.000	1.86	1.24	2.77	0.00
Metachronous	61	25.4%	0%	0%					
Performance status									
WHO 0‒1	77	53.2%	10.2%	1.7%	0.000	0.37	0.24	0.58	0.00
WHO >1	35	9.1%	0%	0%					
Liver metastases									
Yes	46	28.9%	5.9%	0%	0.14				
No	66	47.7%	7.7%	1.9%					
Lung metastases									
Yes	31	40%	13.3%	0%	0.55				
No	81	40%	5%	1.7%					
Bone metastases									
Yes	77	39.5%	7.9%	2%	0.99				
No	35	41.2%	5.9%	0%					
Oligometastatic^c^				0%					
Non	48	26.8%	5.4%		0.006	1.46	0.98	2.17	0.06
Oui	64	53.7%	9.3%	2.3%					

N, Number of patients; OS, Overall Survival; NPC, Nasopharyngeal Carcinoma; WHO, WHO performance status classification; HR, Hazard Ratio; *p*-value, *p*-value of < 0.05 was considered statistically significant.

a Log rank test.

b Cox model.

c Oligometastatic disease: by less than five metastases and/or metastases in only one site.

**Figure 1:**
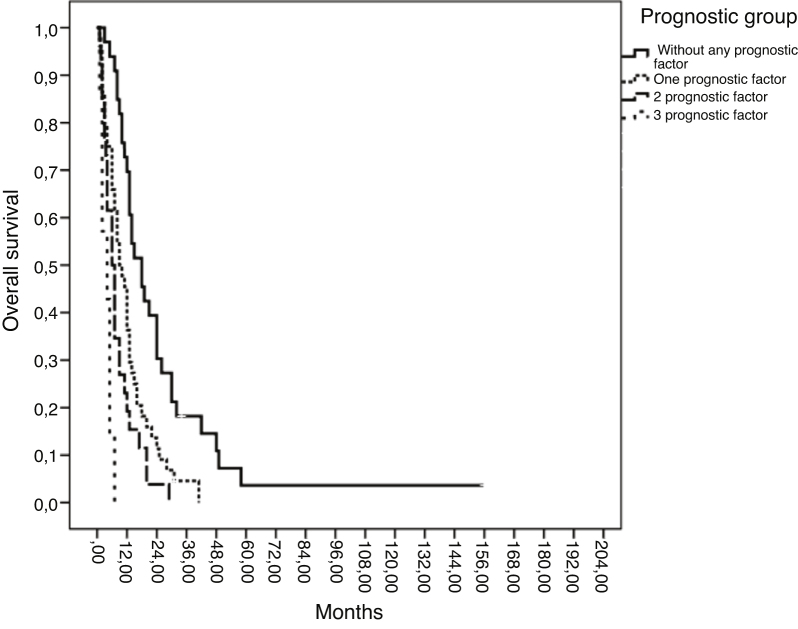
Overall survival of patients according to the number of independent prognostic factors.

According to the number of independent prognostic factors, 4 prognostic groups of patients were found. Patients without any independent prognostic factor had 1 year OS of 69.7% vs. 36.4% for patients who had one prognostic factor (*p* <  0.001). Female patients with metachronous metastasis and poor performance status had a 1 year OS of 0% (Fig. 1).

#### Prognostic factors in patients with synchronous metastaticnasopharyngeal carcinoma

The median OS in smNPC patients was 13 months (1-156). Overall survival at 1 year, 2 years, 3 years and 5 years was respectively 56.9%, 23.5%, 11.8% and 4.7%. In univariate analysis, epidemiological and clinical characteristics associated with poor prognosis were female gender (*p* = 0.005), locally advanced (T4 and/or T3) tumor (*p* = 0.043), poor (WHO > 1) performance status (*p* = 0.002), and non-oligometastatic disease (*p* = 0.045). Therapeutic features that altered the survival of patients weresevere toxicity (G3 and G4) of chemotherapy (*p* = < 0.001), and the lack of irradiation of nasopharyngeal tumor (*p* = 0.043) and of metastatic sites (*p* = 0.046). Taxane-based chemotherapy did not improve OS in smNPC patients (*p* = 0.28).

In multivariate analysis, independent prognostic factors were non-oligometastatic disease (HR = 5.45 [1.5–19.78], *p* =  0.01), severe (Grade 3 or 4) chemotherapy toxicity (HR = 19.1 [4.11–89.1], *p* =  0.001), and the lack of nasopharyngeal irradiation (HR = 0.02 [0.00‒0.073], *p* =  0.0001) and metastatic site irradiation (HR = 0.15 [0.01‒0.32], *p* =  0.007). Table 3 shows studied prognostic factors in univariate and multivariate analysis in the group of smNPC.

**Table 3:** Prognostic factors of synchronous metastatic nasopharyngeal carcinoma patients in univariate and multivariale analysis.

Prognostic factor	Univariate analysis^a^					Multivariate analysis^b^			
	n	OS at 1 year	OS at 3 years	OS at 5 years	*p*-value	HR	Low	High	*p*-value
Gender									
Male	40	62.5%	12.8%	2.6%	0.00	0.14	0.29	0.628	0.11
Female	11	36.4 %	0%	0%					
Mean age									
≤ 50 years	27	57.7%	11.5%	3.8%	0.95				
> 50 years	24	54.2%	8.3%	0%					
T and/or N classification									
T4 and/or N3	17	48.8%	4.7%	2.3%	0.043	0.17	0.02	1.68	0.13
Non T4 and non N3	34	71.4%	42.9%	0%					
Performance status									
WHO 1	39	68.4%	13.2%	2.6%	0.002	0.25	0.06	1.05	0.05
WHO > 1	12	16.7%	0%	0%					
Liver metastases									
Yes	22	45.5%	9.1%	0%	0.43				
No	29	64.3%	10.7%	3.4%					
Lung metastases									
Yes	14	64.3%	14.3%	7.1%	0.36				
No	39	52.8%	8.3%	2.8%					
Bone metastases									
Yes	32	54.8%	9.7%	2.5%	0.97				
No	19	57.9%	5.3%	0%					
Oligometastatic^c^									
No	19	31.6%	10.5%	0%	0.045	5.45	1.5	19.78	0.01
Yes	32	71%	9.7%	3.2%					
Number of cycles of CT									
< 4 cycles	16	56.3%	0%	0%	0.21				
≥ 4 cycles	26	68%	16%	4%					
First line taxane based CT									
Yes	19	66.7%	5.6%	0%	0.28				
No	23	60.9%	13%	4.3%					
First line Anthracyclin-based CT									
Yes	4	50%	0%	0%	0.74				
No	38	64.9%	10.8%	2.7%					
Grade 3 or 4 toxicty of CT									
Yes	22	37.5%	0%	0%	0.000	19.1	4.11	89.1	0.001
No	20	80%	16%	4%					
Nasopharyngeal RT									
Yes	26	76%	20%	8%	0.043	0.02	0.00	0.073	0.0001
No	20	45%	0%	0%					
Metastatic site RT									
Yes	6	57.1%	14.3%	0%	0.046	0.15	0.01	0.32	0.007
No	19	47.7%	0%	0%					

N, Number of patients; OS, Overall Survival; NPC, Nasopharyngeal Carcinoma; WHO, WHO performance status classification; HR, Hazard Ratio; *p*-value, *p*-value of < 0.05 was considered statistically significant; CT, Chemotherapy; RT, Radiation Therapy.

a Log rank test.

b Cox model.

c Oligometastatic disease: by less than five metastases and/or metastases in only one site.

#### Prognostic factors in patients with metachronousmetastatic nasopharyngeal carcinoma

Median survival in mmNPC patients was 7 months (1-41months). OS at 1 year, 2 years, 3 years and 5 years was respectively 24.6%, 6.6%, 0.3% and 0%.

In univariate analysis, epidemiological and clinical characteristics associated with poor prognosis were poor (WHO > 1) performance status (*p* =  0.001), the relapse within 12 months after definitive radiation (*p* =  0.01) and non-oligometastatic disease (*p* =  0.01). Therapeutic features that altered the survival of patients were the lack of taxane-based chemotherapy (*p* =  0.01) as well as the lack of irradiation of metastatic sites (*p* =  0.03).

In multivariate analysis, the poor performance status was an independent prognostic factor in mmNPC patients (HR = 2.71 [1.04–7.06], *p* =  0.04). Table 4 shows studied prognostic factors in univariate and multivariate analysis in mmNPC patients.

**Table 4:** Prognostic factors of metachronous metastatic nasopharyngeal carcinoma patients in univariate and multivariale analysis.

	Univariate analysis^a^					Multivariate analysis^b^			
Prognostic factor	n	OS at 1 year	OS at 3 years	OS at 5 years	*p*-value	HR	Low	High	*p*-value
Gender									
Male	47	23.4%	43%	0%	0.63				
Female	14	28.6 %	0%	0%					
Performance status									
WHO 1	38	36.8%	5.3%	0%	0.001	2.71	1.04	7.06	0.04
WHO > 1	23	4.3%	0%	0%					
Mean age									
≤ 45 years	29	27.6%	3.4%	0%	0.33				
> 45 years	32	21.9%	0%	0%					
Time to relapse									
≤ 12 months	43	14%	2.3%	0%	0.01	0.53	0.26	1.11	0.94
< 12 months	18	50%	5.6%	0%					
Liver metastases									
Yes	24	12.5%	0%	0%	0.06				
No	37	32.4%	2.7%	0%					
Lung metastases									
Yes	17	17.6%	0%	0%	0.76				
No	44	27.3%	0%	0%					
Bone metastases									
Yes	44	27.3%	0%	0%	0.32				
No	17	17.6%	5.9%	0%					
Oligometastatic^c^									
No	29	13%	0%	0%	0.01	0.78	0.35	1.76	0.54
Yes	32	34.4%	3.1%	0%					
First line taxane based CT									
Yes	16	52.6%	0%	0%	0.01	0.53	0.23	1.224	0.13
No	29	15.4%	0%	0%					
First line Anthracyclin-based CT									
Yes	26	40%	8%	0%	0.12				
No	19	21.1%	0%	0%					
RT of metastatic sites									
Yes	9	70%	10%	0%	0.03	0.61	0.24	1.59	0.31
No	31	22.6%	0%	0%					

N, Number of patients; OS, Overall Survival; NPC, Nasopharyngeal Carcinoma; WHO, WHO performance status classification; HR, Hazard Ratio; *p*-value, *p*-value of < 0.05 was considered statistically significant; CT, Chemotherapy; RT, Radiation Therapy.

a Log rank test.

b Cox model.

c Oligometastatic disease: by less than five metastases and/or metastases in only one site.

### Long-term survivors

We found 6 long-term survivors in our study (5 patients with smNPC and 1 patient mmNPC). All smNPC long-term survivors’ patients had received nasopharyngeal radiation therapy and three among five patients received radiation therapy to metastatic site. The long-term survivor of the mmNPC group of patients had a relapse after 12 months, developed an oligometastatic disease and received radiation therapy to the metastatic site.

## Discussion

This study provided several notable finding concerning prognostic factors. First, patients with oligometastatic disease had the best survival. Second, locoregional treatment of primitive NPC improved survival in smNPC patients who responded to induction chemotherapy. Third, local irradiation of metastatic sites improved survival of metastatic NPC patients. Finally, severe chemotherapy toxicity (Grade 3 or 4) altered survival among smNPC patients.

Patients with oligometastatic disease had better OS in both groups in our study, however, oligometastatic disease has been defined differently in several studies.^12^, ^13^, ^14^, ^15^ In a retrospective analysis of 263 patients with stage IVc NPC,^12^ Tian subdivided patients into two groups of metastatic disease which were M1a (single organ or 1–5 lesions) and M1b (multiple organ or > 5 lesions). The 5‐year OS rates for M1a disease and M1b disease were 38.7% and 7.0%, respectively (*p* <  0.01).

In our study, nasopharyngeal radiation therapy after stable disease or partial/complete response to chemotherapy was an independent prognostic factors in smNPC patients (HR = 0.02 (0.00‒0.073), *p*  = 0.0001). Nasopharyngeal radiation therapy in synchronous metastatic NPC patients has been debated due to the short life expectancy and risk of serious late complications. However, many retrospective studies showed an improvement of survival, tumor local control, patient’s quality of life by reducing necrosis, bleeding and severe headaches.^16^, ^17^

In a recent phase III trial,^18^ 126 patients with stage IVc NPC were randomized, after a primary complete or partial response to induction CT, to receive either CT or CT and nasopharyngeal RT. Chemotherapy plus RT in patients who responded to this treatment significantly improved OS (40.2 vs. 24.5 months, HR = 0.45, 95% CI 0.25‒0.80; *p*  = 0.007) in primary metastatic NPC.

Local therapy of metastatic sites showed an improvement, in our study, of the OS of patients with smNPC in multivariate analysis (HR = 0.15 [0.01‒0.32], *p* = 0.0007) and of the OS of patients with mmNPC in univariate analysis (*p*  = 0.03). In our study, radiation therapy was used as a local therapy for oligometastatic patients. Local treatments of metastatic NPC were invasive in many studies, such as hepatectomy or lung metastases resection or less invasive such as Transcatheter hepatic Artery Chemoembolization (TACE) or Radiofrequency Ablation (RA).^14^, ^19^, ^20^

Local therapy of metastatic sites was associated with an improvement in survival and in the treatment outcome in several retrospective studies.^15^, ^20^, ^21^, ^22^ In a retrospective cohort of 448 patients who developed distant metastases after initial treatment, Liang et al.^23^ found an improvement of the 3 year OS in patients who received local treatment of distant metastasis, compared with patients who did not (48.8% vs. 33.8%, *p*  = 0.001). Due to the lack of phase III trials, it is still unclear which patients may benefit the most from local therapies to metastatic sites. In a large multicenter population study of 3 cohorts of 462 metastatic NPC patients, the local treatment of metastatic lesion was not associated with an improvement of OS in patients undergoing CT and locoregional RT, even in oligometastatic patients without liver involvement.^14^

Palliative systemic CT is the major treatment modality for metastatic NPC patients.^24^, ^25^ The combination of cisplatin and 5-fluorouracil (PF) has been one of the standard first-line therapies in metastatic NPC patients.^26^ The association of docetaxel to PF (TPF) enables an objective response of 73%.^27^ In our study, taxanes-based first-line chemotherapy improved OS in metachronous metastatic patients in univariate analysis (*p*  = 0.01) however, Grade 3 or 4 chemotherapy toxicity altered significantly OS in univariate and multivariate analysis. This concludes that CT protocols should be adjusted for every patient according to age and comorbidities in order to prevent treatment related toxicities.

This study has several limitations. First, it is a retrospective study, and second, we failed to include in our analysis other prognostic factors such as hemoglobin, LDH, leucocytes and albumin due to the lack of available data in medical records. The serum Epstein-Barr Virus (EBV) DNA level has been demonstrated to be an important prognostic factor among patients with metastatic NPC.^28^

## Conclusion

Oligometastatic patients had better survival. Metastatic NPC should not have aggressive treatment that can lead to Grade 3 or 4 toxicity. Synchronous metastatic patients should receive chemotherapy followed by nasopharyngeal radiation therapy if patients are responding well, since it improves outcome significantly. In addition, irradiation to a metastatic site in oligometastatic patients should be considered since it leads to an improvement of survival. Further phase III randomized trials are required in order to assess which patients are best candidates for aggressive treatments, including metastases local treatment and nasopharyngeal radiation.

## Conflicts of interest

The authors declare no conflicts of interest.
